# Deep learning methods improve genomic prediction of wheat breeding

**DOI:** 10.3389/fpls.2024.1324090

**Published:** 2024-03-04

**Authors:** Abelardo Montesinos-López, Leonardo Crespo-Herrera, Susanna Dreisigacker, Guillermo Gerard, Paolo Vitale, Carolina Saint Pierre, Velu Govindan, Zerihun Tadesse Tarekegn, Moisés Chavira Flores, Paulino Pérez-Rodríguez, Sofía Ramos-Pulido, Morten Lillemo, Huihui Li, Osval A. Montesinos-López, Jose Crossa

**Affiliations:** ^1^ Departamento de Matemáticas, Centro Universitario de Ciencias Exactas e Ingenierías (CUCEI), Universidad de Guadalajara, Guadalajara, Jalisco, Mexico; ^2^ International Maize and Wheat Improvement Center (CIMMYT), Texcoco, Estado. de México, Mexico; ^3^ Instituto de Investigaciones en Matemáticas Aplicadas y Sistemas (IIMAS), Universidad Nacional Autónoma de México (UNAM), Ciudad Universitaria, Ciudad de México, Mexico; ^4^ Estudios del Desarrollo Rural, Economía, Estadística y Cómputo Aplicado, Colegio de Postgraduados, Texcoco, Estado de México, Mexico; ^5^ Department of Plant Science, Norwegian University of Life Science (NMBU), Ås, Norway; ^6^ 6State Key Laboratory of Crop Gene Resources and Breeding, Institute of Crop Sciences and CIMMYT China Office, Chinese Academy of Agricultural Sciences (CAAS), Beijing, China; ^7^ Facultad de Telemática, Universidad de Colima, Colima, Colima, Mexico

**Keywords:** GBLUP model, genomic prediction, multi-modal deep learning model, machine learning methods, relationship matrices

## Abstract

In the field of plant breeding, various machine learning models have been developed and studied to evaluate the genomic prediction (GP) accuracy of unseen phenotypes. Deep learning has shown promise. However, most studies on deep learning in plant breeding have been limited to small datasets, and only a few have explored its application in moderate-sized datasets. In this study, we aimed to address this limitation by utilizing a moderately large dataset. We examined the performance of a deep learning (DL) model and compared it with the widely used and powerful best linear unbiased prediction (GBLUP) model. The goal was to assess the GP accuracy in the context of a five-fold cross-validation strategy and when predicting complete environments using the DL model. The results revealed the DL model outperformed the GBLUP model in terms of GP accuracy for two out of the five included traits in the five-fold cross-validation strategy, with similar results in the other traits. This indicates the superiority of the DL model in predicting these specific traits. Furthermore, when predicting complete environments using the leave-one-environment-out (LOEO) approach, the DL model demonstrated competitive performance. It is worth noting that the DL model employed in this study extends a previously proposed multi-modal DL model, which had been primarily applied to image data but with small datasets. By utilizing a moderately large dataset, we were able to evaluate the performance and potential of the DL model in a context with more information and challenging scenario in plant breeding.

## Introduction

Wheat holds immense importance globally as a vital crop that serves as a staple food source for a significant portion of the world’s population (Poland et al., 2012). It is cultivated in diverse agroclimatic regions and plays a critical role in ensuring global food security (FAO, 2021). The primary objective of wheat breeding programs is to develop superior varieties with enhanced traits such as higher yield potential, improved disease resistance, and better end-use quality. To expedite the breeding process and maximize genetic progress, genomics selection (GS) has emerged as a powerful tool (Crossa et al., 2017). In this context, genomic prediction has been extensively studied to enhance the efficiency of wheat breeding programs. It incorporates genomic relationship matrices to estimate the genetic variance and predict breeding values based on marker information.

Researchers have developed various statistical models to predict the performance of wheat lines based on genomic data. One fundamental and widely used model in genomic prediction is the Genomic Best Linear Unbiased Prediction (GBLUP) model, due in part to its simplicity and effectiveness in accounting for genetic relationships and accurately predict breeding values. GBLUP has demonstrated promising results in predicting complex traits in wheat, including yield, disease resistance, and quality attributes (Heffner et al., 2011; Poland et al., 2012; Rutkoski et al., 2016).

In recent years, deep learning models have gained attention for genomic prediction tasks in wheat. These models leverage the power of neural networks to learn complex patterns and relationships in genomic data (Crossa et al., 2017; Montesinos-López et al., 2018). The convolutional neuronal and the multilayer perceptron networks are the most common architecture applied in GS (Jiang and Li, 2020), and to reduce the number of weights to estimate during the training process more often a compressed version of the matrix of genomic relationship is used to feed the network instead of directly using the thousands of single nucleotide polymorphisms (SNP) available (Montesinos-López et al., 2018, Montesinos-López et al., 2021).

More recently, multi-modal deep learning models have emerged as an alternative that leverages multiple data modalities to improve prediction and analysis tasks (Liu et al., 2018). These models integrate multiple types of data inputs, such as genomic, phenotypic, and image environmental data, to improve prediction accuracy and robustness. By combining information from various sources, multi-modal models capture the interactions and correlations between different data modalities, leading to more accurate predictions and a better understanding of the underlying genetic architecture (Rahate et al., 2022).

Multi-modal deep learning has been explored and applied in diverse research fields, including the field of healthcare (Huang et al., 2020; Venugopalan et al., 2021; Kline et al., 2022; Stahlschmidt et al., 2022), agriculture (Danilevicz et al., 2021; Garillos-Manliguez and Chiang, 2021; Zhou et al., 2021), material sciences (Muroga et al., 2023), natural language processing (Morency and Baltrušaitis, 2017; Zadeh et al., 2018), social media analysis (Balaji et al., 2021; Chandrasekaran et al., 2021), robotics and autonomous perception (Melotti et al., 2020; Duan et al., 2022).

For an early overview on deep multi-modal learning models see Ngiam et al. (2011) and Srivastava and Salakhutdinov (2012), and for a survey of recent advances in multi-modal machine learning see Ramachandram and Taylor (2017); Summaira et al. (2021) and Jabeen et al. (2023). In wheat genomic prediction, multi-modal deep learning models have been explored and applied as a promising approach (Kick et al., 2023; Montesinos-López et al., 2023). These studies have demonstrated the potential of multi-modal deep learning in enhancing the accuracy of genomic prediction for wheat traits.

Based on the previous considerations on how DL can be employed for genomic prediction in this study we follow a similar network structure as the previous study of Montesinos-López et al. (2023), up to the output layer. However, instead of directly combining the final outputs of individual networks from each modality to create the final output, we introduced an additional layer under a multi-layer perceptron network. This network has a similar architecture to the individual networks in each modality but with its own set of hyperparameters, which are also part of the tuning process. Furthermore, this study involves a moderately large dataset (4,464 wheat lines), allowing for a comprehensive evaluation of prediction accuracy. We compared the performance of our multi-modal deep learning model with the powerful GBLUP model, widely used in this field. This comparison enables us to assess the effectiveness of the multi-modal approach and its potential for enhancing genomic prediction accuracy in this specific context.

## Materials and methods

### Phenotypic data

The phenotypic data corresponds to the measurement of five traits (Yield, Germination, Heading, Height, and Maturity) in 4,464 wheat lines grown during the 2021/2022 crop season at the Norman E Borlaug Experiment Station, Ciudad Obregon (27°20′ N, 109°54′ W), Sonora, Mexico. The complete set of lines was tested under four different environments: (1) Beds with five irrigations (B5IR): genotypes were grown on raised beds with about 500 mm of available water and optimal sowing date during late November–early December, (2) Beds with two irrigations (B2IR): genotypes were grown on raised beds with about 250 mm of available water and optimal sowing date, (3) Bed Drought-Drip stress (BDRT): genotypes were grown on raised beds with about 120 mm of available water and optimal sowing date, and (4) Bed late heat stress (BLHT): genotypes were grown on raised beds with about 500 mm of available water and late sowing date (mid-February). Yield was measured in all environments, while Germination, Heading, Height and Maturity were determined in three out of four (B5IR, B2IR, and BDRT). Recently this data set was employed by Montesinos-López et al. (2023) for assessing the benefit of applying sparse phenotype field trials for genomic prediction at early testing generation of the population improvement (occurring at F_4_ or F_5_)>.

### Genotypic data

The genotypic information comprised a total of 18,239 SNP markers. Genotyping was performed using the Genotyping-by-Sequencing (GBS) method, employing an Illumina HiSeq2500 sequencer at Kansas State University (Poland et al., 2012). Quality control was conducted using TASSEL v5.0 software (https://tassel.bitbucket.io). Raw data underwent filtration based on a minor allele frequency (MAF) cut-off of less than 5% and a missing data threshold of less than 50%. Subsequently, the HapMap file was converted into a numerical matrix to enable compatibility with the genomic prediction software. For the numerical representation, TASSEL assigned a value of 1 for homozygous major alleles, 0 for homozygous minor alleles, and 0.5 for heterozygous genotypes. To align the numerical matrix with the analysis tools utilized, substitution coding was applied, substituting the values with -1, 1, and 0, respectively. Finally, mean imputation was employed to address any missing values in the numerical matrix.

### Statistical models

#### Bayesian GBLUP model

One of the statistical models used assumes that each response variable follows the relation:


(1) $$Y_{ij}=\mu +E_{i}+{ɡ}_{j}+ɡE_{ij}+{\text{ϵ}}_{ij}$$

where $Y_{ij}$ is the response variable for line *j* in environment *i*, *μ* is the general mean, $E_{i}$ are the fixed effects of environment, ${ɡ}_{j}$ and $ɡE_{ij}$ are the random effects of lines and random interaction effects of environment and line, respectively, and $\epsilon_{ij}$ are the random error terms assumed to be independents normal random variables with mean 0 and variance $\sigma_{\epsilon }^{2}$ . In addition, the random effects of lines and random genotype by environment interaction are assumed independently each other with the following distribution: $ɡ={\left({ɡ}_{1},\ldots ,{ɡ}_{J}\right)}^{T}∼N_{J}\left(0_{J},\sigma_{ɡ}^{2}G\right)$ and $ɡE={\left(E{ɡ}_{11},\ldots ,E{ɡ}_{IJ}\right)}^{T}∼N_{IJ}\left(0_{IJ},\sigma_{Eɡ}^{2}\left(I_{I}⊗G\right)\right)$ with $0_{J}$ and $I_{I}$ the null vector of size J and the identity matrix of dimensions I× I , and ⊗ the Kronecker product.

A Bayesian estimation of these models was performed using a flat prior for the general mean and the fixed effects. For the variance components ( $\sigma_{\epsilon }^{2}$ , $\sigma_{ɡ}^{2}$ and $\sigma_{Eɡ}^{2}$ ) a scale inverse chi-squared distribution was employed. The model was implemented using the BGLR R package (Pérez-Rodríguez and de los Campos, 2014) with the default hyperparameter values.

#### DL model

The same information used in Equations 1 was employed to make predictions under the following multi-modal deep learning model (DL) with single output (Ouyang et al., 2014; Ramachandram and Taylor, 2017):


(2) $$Y_{ij}=f\left(x_{ij};\text{W}\right)=f_{O}\left(w_{0}^{\left(O\right)}+x_{ij}^{*\left(L\right)T}{\text{w}}_{1}^{\left(O\right)}\;\right)$$

where $f_{O}$ is the output activation function with associated weights $w_{0}^{\left(O\right)}$ and $w_{1}^{\left(O\right)}$ . $x_{ij}^{*\left(L\right)T}$ is the transpose of the vector with the neurons of last hidden layer ( $x_{ij}^{*\left(L\right)}$ ) for a multilayer perceptron (MLP) neural network with *L* hidden layers, each layer with $N^{\left(l\right)}$ neurons and activation function $f_{l}(l=1,.,L)$ , that use as input the concatenated outputs of the *Q* separately neural networks apply to each modality. That is, $x_{ij}^{*\left(L\right)}$ is computed recursively from:


$$\begin{array}{l} {\text{x}}_{ij}^{*\left(l\right)}={\left[{\text{x}}_{ij1}^{*\left(l\right)T},\ldots ,{\text{x}}_{ijN^{\left(l\right)}}^{*\left(l\right)T}\right]}^{T}=\;{\left[f_{l}\left(z_{ij1}^{*\left(l\right)}\right),\ldots ,f_{l}\left(z_{ijN^{\left(l\right)}}^{*\left(l\right)}\right)\right]}^{\text{T}}\\ ={\left[f_{l}\left(w_{01}^{\left(l\right)}+{\text{x}}_{ij}^{*\left(l-1\right)T}{\text{w}}_{11}^{\left(l\right)}\right),\ldots ,f_{l}\left(w_{0N^{\left(l\right)}}^{\left(l\right)}+{\text{x}}_{ij}^{*\left(l-1\right)T}{\text{w}}_{1N^{\left(l\right)}}^{\left(l\right)}\right)\right]}^{T} \end{array}$$

where $W_{k}^{\left(l\right)}={\left[w_{1}^{\left(l\right)},\ldots ,w_{N^{\left(l\right)}}^{\left(l\right)}\right]}^{T}$ is the matrix of weights for layer l with $w_{k}^{\left(l\right)}={\left[w_{0k}^{\left(l\right)},w_{1k}^{T\left(l\right)}\right]}^{T}$ for $\;k=1,.,N^{\left(l\right)}$ . Here $x_{ij}^{*\left(0\right)}$ is defined as $x_{ij}^{*\left(0\right)}={\left[x_{ij\left(1\right)}^{\left(L_{1}\right)T},\ldots ,x_{ij\left(Q\right)}^{\left(L_{Q}\right)T}\right]}^{T}$ with $x_{ijq}^{\left(L_{q}\right)}\;$ denoting the transpose of the vector $x_{ij\left(q\right)}^{\left(L_{q}\right)}$ that contain the outputs of the last hidden layer of the *q*-th MLP neural network (with $L_{q}$ hidden layers, each layer with $N_{q}^{\left(l\right)}$ neurons and activation function $f_{l}^{\left(q\right)}$ , $l=1,.,L_{q}$ ) corresponding to the q -th modality ( q=1,…,Q ), which in turn are computed recursively as:


$${\text{x}}_{ij\left(q\right)}^{\left(l\right)}={\left[x_{ij1\left(q\right)}^{\left(l\right)},\ldots ,x_{ijN_{q}^{\left(l\right)}\left(q\right)}^{\left(l\right)}\right]}^{T}={\left[f_{l}^{\left(q\right)}\left(z_{ij1\left(q\right)}^{\left(l\right)}\right),\ldots ,f_{l}^{\left(q\right)}\left(z_{ijN_{q}^{\left(l\right)}\left(q\right)}^{\left(l\right)}\right)\right]}^{T}$$

where $z_{ijk\left(q\right)}^{\left(l\right)}=w_{0k\left(q\right)}^{\left(l\right)}+x_{ij\left(q\right)}^{\left(l-1\right)T}w_{1k\left(q\right)}^{\left(l\right)}$ , $k=1,\ldots ,N_{q}^{\left(l\right)}$ , are linear transformations of the $N_{q}^{\left(l-1\right)}$ neurons in layer l−1 that define the neurons in layer l after applying the activation function $f_{l}^{\left(q\right)}$ , $x_{ijkq}^{\left(l\right)}=f_{l}^{\left(q\right)}\left(z_{ijkq}^{\left(l\right)}\right)$ , $W_{k\left(q\right)}^{\left(l\right)}={\left[w_{1\left(q\right)}^{\left(l\right)},\ldots ,w_{N^{\left(l\right)}\left(q\right)}^{\left(l\right)}\right]}^{T}$ is the matrix of weights for the hidden layer *l* ( $l=1,.,L_{q}$ ) for the *q*-th neural network, $w_{k\left(q\right)}^{\left(l\right)}={\left[w_{0k\left(q\right)}^{\left(l\right)},w_{1k\left(q\right)}^{T\left(l\right)}\right]}^{T}$ for $\;k=1,.,N_{q}^{\left(l\right)}$ , and $x_{ij\left(q\right)}^{\left(0\right)}=x_{ij\left(q\right)}$ are the inputs corresponding to *q*-th modality.

In the implemented models, all applied deep learning models are versions of Equation 2 that utilized a stacked residual network (ResNet) composed of 2 sequence layers (He et al., 2016). These were implemented with library TensorFlow in Python software, using a Batch_size value equal to 32, 48 epochs and the Adam optimizer (a stochastic gradient descend method to minimize the penalized loss function in DL) and using callback options of the fit keras function and specifying an adaptative exponential decay learning scheduler.

In all, for each modality (type of input) the number of units after the second hidden layer was equal to half of the units in the preceding layer, for example, for the neural network for the *q*-th modality,


$$N_{q}^{\left(l\right)}\;=\;\lfloor \frac{N_{q}^{\left(1\right)}}{2^{l-1}}\rfloor ,\;l\;=\;2,\;.\;.\;.\;,\;L_{q}$$

where *x* denotes the largest integer less than *x*, and $N_{q}^{\left(1\right)}$ is the required number of units for the first hidden layer. Similarly, for the multilayer perceptron network after concatenating the outputs of the *Q* individual MLP neuronal networks, for a specified neurons in its first layer ( $N^{\left(1\right)}$ ), the neurons of the latter layers was taken as $N^{\left(l\right)}\;=\;\lfloor \frac{N^{\left(1\right)}}{2^{l-1}}\rfloor ,\;l\;=\;2,\;.\;.\;.\;,\;L$ .

**Figure 1:**
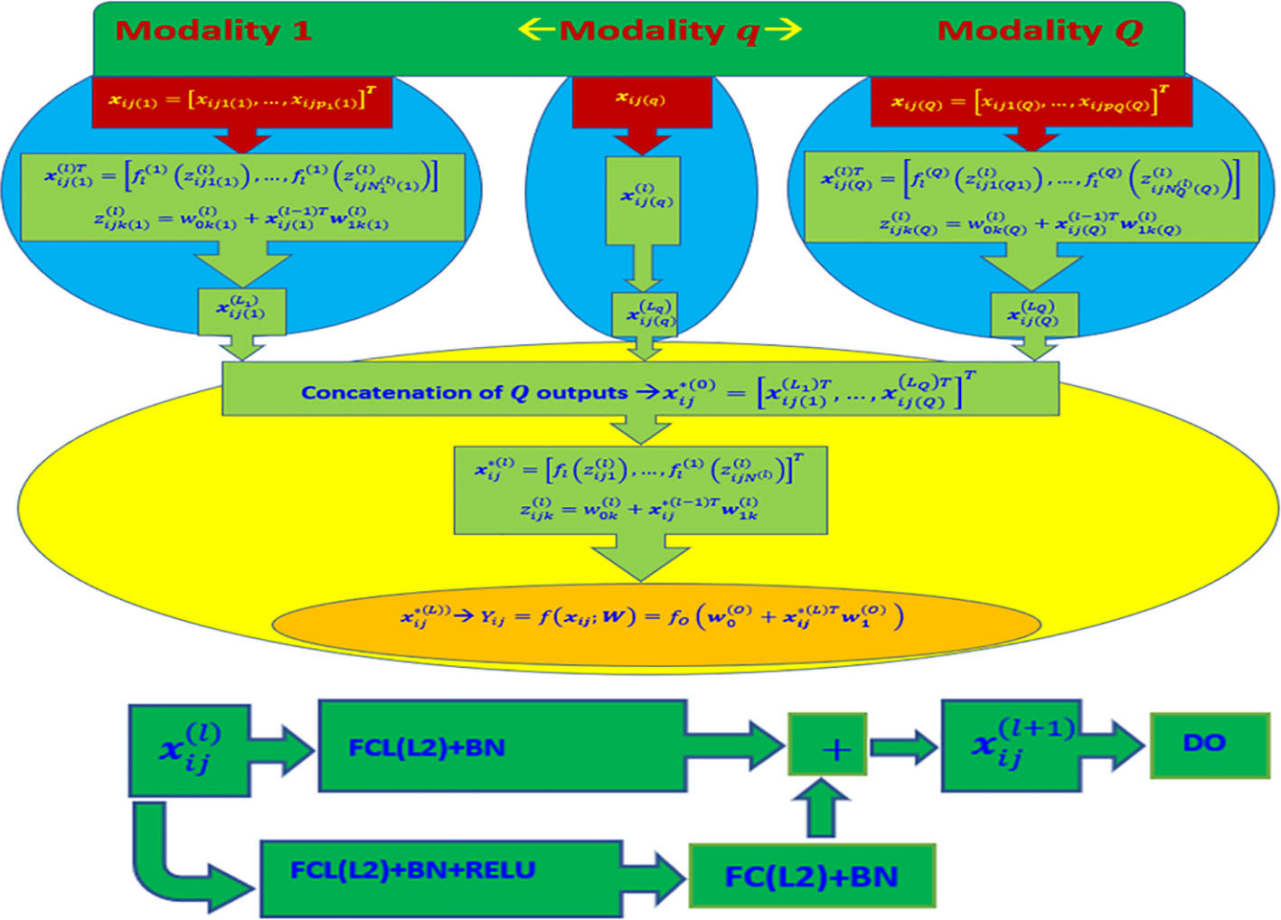
Top diagram: Multi-modal deep learning model (DL) with Q modalities (types of input). Bottom diagram: Stacked Residual Network (ResNet) composed of two sequential dense layers (FCL) applied in each MLP Neural Network. FCL(L2) + BN + ReLU denotes the successive application of a fully connected layer (FCL) with L2 regularization, batch normalization layer, and a ReLU activation function. Similarly, FCL(L2) + BN indicates the application of a fully connected layer with L2 regularization and batch normalization, while “DO” indicates the application of dropout regularization. The final output is produced by using the concatenated outputs of the Q networks as input to another MLP Neural Network. The output layer of this network consists of one neuron with a linear activation function and L2 regularization for its weights (concatenated outputs of all Q MLP Neural Networks + FCL + L2).

The rectified linear unit (ReLU) activation function was utilized in all hidden layers of the model, except for the output layer. For the output layer, a linear activation function was employed, assuming the conditional distribution of each trait follows a normal distribution. After each dense layer and prior to applying the activation function, a batch normalization layer was inserted. This layer help in approximately standardizing the outputs, ensuring a mean close to 0 and a standard deviation close to 1. For more detailed information, please refer to **Figure 1**.

For the training process, we employed an inner 10-fold cross-validation strategy. To expedite the training, only two out of the ten folds are utilized for validation. An early stopping rule is implemented through the callback option. The rule specifies monitoring the ‘loss’ function, with a mode of ‘min’ and a patience of ‘Pat’. This rule checks whether the loss function on the training data stops decreasing at the end of each epoch. If it continues for an additional ‘Pat’ epochs, the training is halted.

To mitigate overfitting, dropout and L2 regularization were incorporated at each hidden layer, while only L2 regularization applied to the output layer. L2 regularization penalizes the loss function (e.g., sum of squared error loss) by adding the sum of squared weights multiplied by a regularization parameter (λ). This parameter controls the extent to which the weights are shrunk toward zero, reducing the model’s complexity and preventing excessive fitting to the training data. Dropout involves randomly setting a fraction of the weights to 0 at each training step.

Hyperparameters tuned in the experiment included learning decay (wd), patience values (Pat), dropout rate (DO), and regularization parameters (λ). The optimization of these hyperparameters was performed using the bayes_opt library with 50 iterations. The objective was to find the combination of hyperparameter values that minimized the mean squared error on the validation set. **Table 1** provides a complete list of the hyperparameters and their corresponding search space.

**Table 1:** Hyperparameters of the DL model and their respective domain space.

Hyperparameter	Notation	Bounds
Hidden layers for the MLP NN for each modality Hidden layer for the MLP after concatenating the outputs of the NN of the 3 modalities	$L_{1},\;L_{2}$ and $L_{3}$	(1,4), (1,6) and (1,6)
	L	(0,4)
Number of neurons for the first layer in each modality Number of neurons for the first layer in the MLP after concatenating the outputs of the NN of the 3 modalities	$N_{1}^{\left(1\right)},\;\;N_{2}^{\left(1\right)},\;N_{3}^{\left(1\right)}$	(0,128) , (1,1024) and (1, 1024)
	${\text{N}}^{\left(1\right)}$	(0,200)
Regularization parameter for L2	λ	(1e-8,1e-2)
Dropout	DO	( $1\times 10^{-4}$ ,0.5)
Log weight decay	lwd=ln(wd)	$\left(\ln\left(4\times 10^{-5}\right),\ln\left(4\times 10^{-1}\right)\right)$
Patience	Pat	(0,128)
Log learning rate	llr=ln(lr)	$\left(\ln\left(1\times 10^{-8}\right),l\text{n}\left(1\times 10^{-2}\right)\right)$

The models were executed on a single computer node with 32 GB of RAM and 16 cores, together with a 20 GB GPU, and the experiments were conducted using Python version 3.8.10 and TensorFlow 2.11.0. On average, training each time a DL model with the specified characteristics described in the paper took approximately between 8 and 15 hours. In subsequent references within this manuscript, DL will be used to denote the specific deep learning (DL) model given in Equation 2, except in the LOEO evaluation where only the line effect is used.

Specifically, for the 5-fold cross-validation (5FCV) strategy described in the next section, the multi-modal DL Equation 2 was trained with 3 modalities corresponding to the information of the matrix design of environment ( $X_{E}$ ), the genotype information $(X_{L}=Z_{L}L_{G}$ ) and the environment-genotype interaction information ( $X_{EL}=Z_{EL}L_{EG}$ ), where $Z_{L}\;$ and $Z_{EL}$ are the matrix design of lines and the matrix design of the environment-line interaction, and $L_{G}$ and $L_{EG}$ are respectively the upper triangular part of the Cholesky decomposition of the genomic relationship matrix $G\;\left(G=L_{G}^{T}L_{G}\right)$ and the upper triangular part of the Cholesky decomposition of the “environment-genomic” relationship matrix $G_{EG}=I_{I}⊗G\;\left(G_{EG}=L_{EG}^{T}L_{EG}\right)$ .

To evaluate the DL models for predicting the performance of an entire environment using the lines from all other environments (LOEO), the same DL model was employed. However, in this case, only the information of the matrix design of environment ( $X_{E}$ ) and the genotype information were utilized as inputs. As a result, in the first predictor (GID) the DL is reduced to a single-modal DL model.

### Assessment of prediction accuracy

Two strategies were used to evaluate and compare the models’ predictive performance. The first strategy, 5FCV, involved dividing the dataset into five balanced subsets. Four subsets were used for training the model, while the remaining subset was reserved for testing. This process was repeated, ensuring each subset served as the testing set once. The model’s performance was assessed by calculating the average Normalized Root Mean Squared Error (NRMSE) and Pearson’s correlation coefficient across all five partitions. The standard deviation was also computed to judge performance variability.

The second strategy, LOEO, is focused on predicting an entire environment using data from the other environments as training. During training, the models excluded the effects of environment (*E*) and the interaction between environment and lines (*Eg*). NRMSE and Pearson’s correlation coefficient were calculated for each predicted environment separately, allowing a detailed evaluation of the model’s performance in predicting specific environments.

By employing these strategies, the models’ predictive accuracy was assessed using NRMSE and Pearson’s correlation coefficient. The 5FCV approach provided an overall performance evaluation across the five cross-validation partitions, while LOEO enabled the evaluation of performance in individual environments.

## Data availability

The phenotypic and genomic wheat data employed in this study can be downloaded from the following link https://hdl.handle.net/11529/10548813 (Montesinos-López et al., 2023).

## Results

The results are provided in three sections. First, for evaluating the prediction performance under tested lines in tested environments under a 5FCV, second, under tested lines in untested environments under the LOEO strategy and third, a summary of the hyperparameter values used in the trained models.

### Tested lines in tested environments under a 5FCV strategy

**Figure 2:**
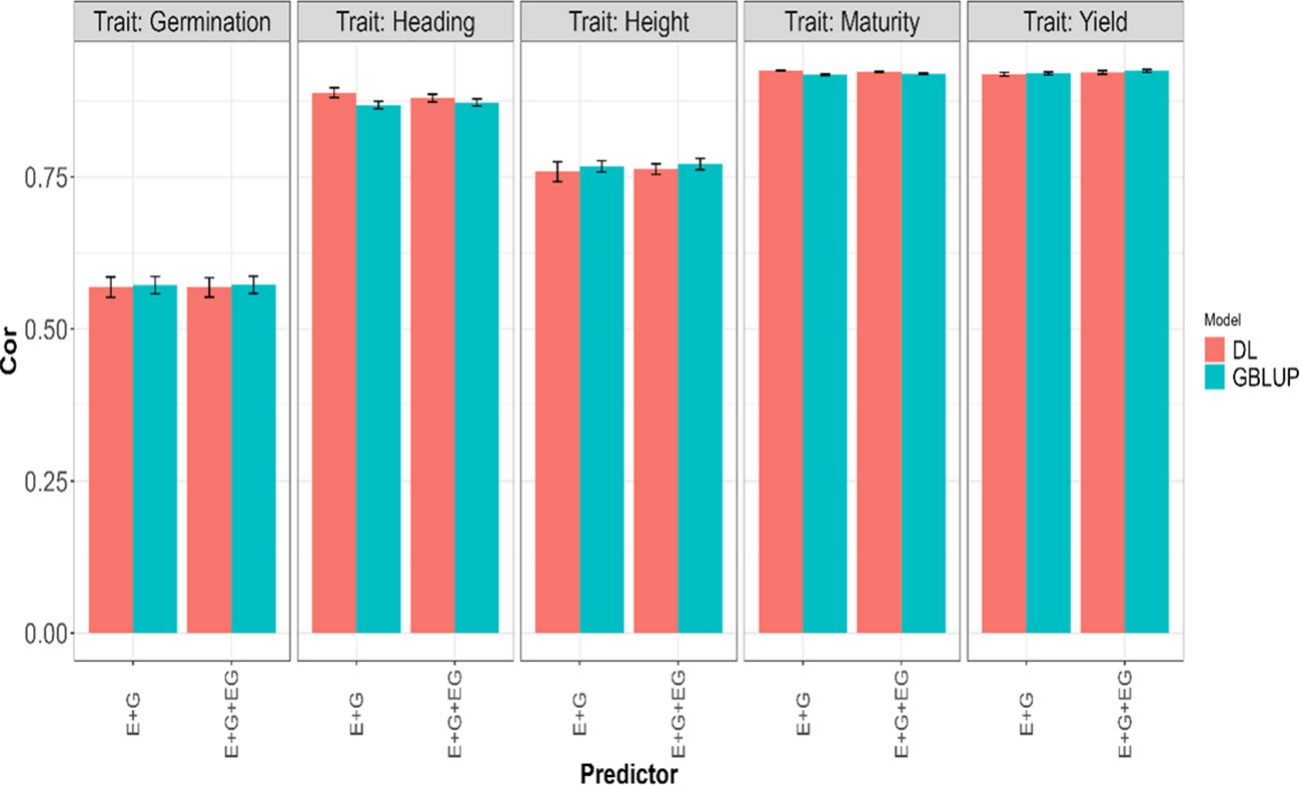
Average Pearson’s correlation (Cor) across five-fold cross-validation for each of the five traits (Germination, Heading, Height, Maturity, and Yield) for GBLUP and deep learning (DL) models using two predictors (E+G and E+G+GE). The limits of the vertical lines in each bar indicate the average minus and plus one standard deviation (SD) values of Cor obtained across folds. E, G, and GE represent the environment, lines, and environment-lines interaction effects, respectively.

**Figure 3:**
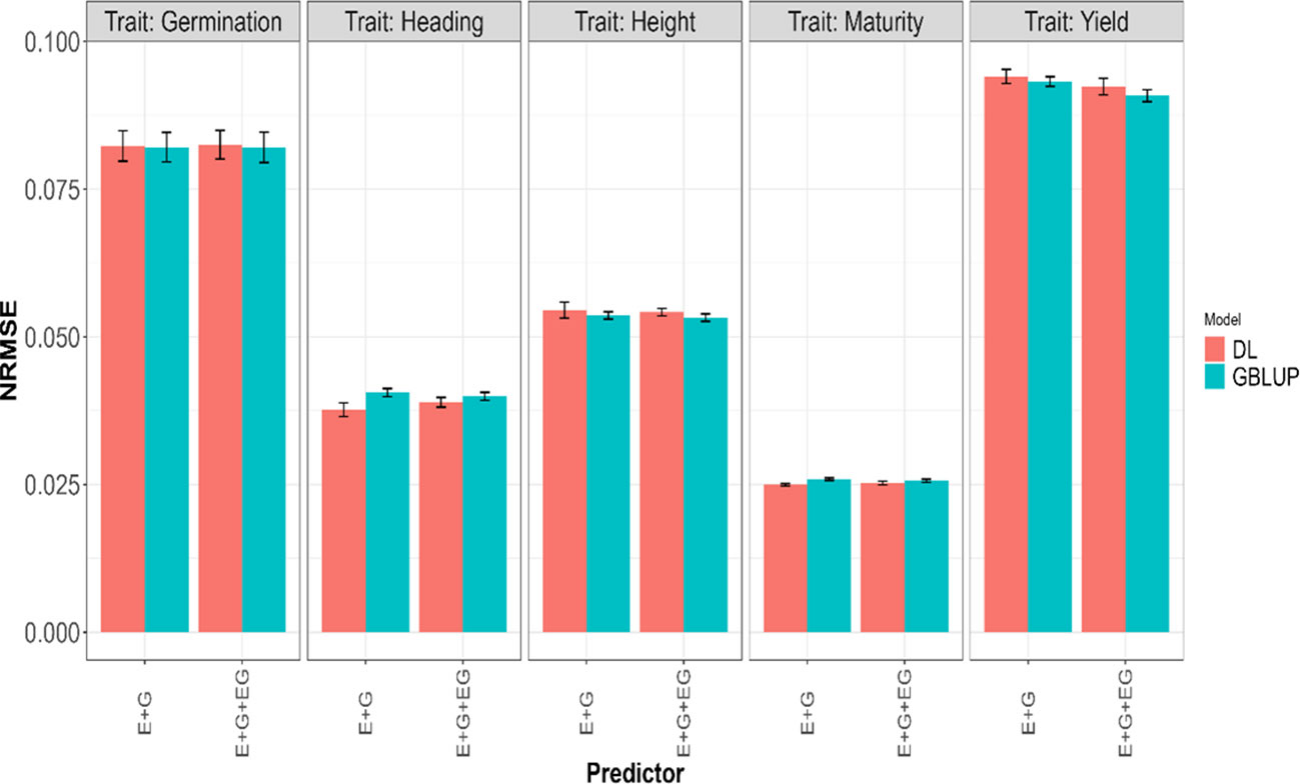
Average normalized mean squared error (NRMSE) across five-fold cross-validation for each of the five traits (Germination, Heading, Height, Maturity, and Yield) for GBLUP and deep learning (DL) models using two predictors (E+G and E+G+GE). The limits of the vertical lines in each bar indicate the average minus and plus one standard deviation (SD) values of Cor obtained across folds. E, G, and GE represent the environment, lines, and environment-lines interaction effects, respectively.

The fitted models for each of the four traits separately included the GBLUP Equation 1 and the deep learning Equation 2, along with sub-models of these primary models. Specifically, the first assessment of these models regarding its genomic prediction ability was conducted using the 5FCV strategy with the predictors E+G+GE and E+G . The results are presented in **Table 2** with the first, second and third columns indicating the model (GBLUP or DL), the trait and the predictor, respectively, and the last two columns the average and standard deviations values of the evaluated metrics (NRMSE and Cor). The results are also displayed in **Figures 2** and **3**. From **Table 2**, it can be observed that the GBLUP model performed best on average under the two evaluated metrics for three out of the five studied traits: Yield, Height, and Germination. The DL models showed an average NRMSE between 0.27% and 1.76% higher than the corresponding GBLUP models. However, the difference in performance was less pronounced for the Germination trait. In terms of the average correlation (Cor), the GBLUP model had values between 0.15% and 1.13% higher than those observed with the DL models. With this metric, the difference in performance was less pronounced for the Yield trait.

**Table 2:** Average normalized root mean squared error of prediction (NRMSE) and average Pearson’s correlation (Cor) in a 5-fold cross-validation strategy when predicting each one of the five traits (Yield, Maturity, Height, Heading and Germination) with GBLUP and DL models using E+G and E+G+EG as predictors.

Model	Trait	Predictor	NRMSE (SD)	Cor (SD)
GBLUP	Yield	E+G	0.0932(0.0008)	0.9203(0.0024)
GBLUP	Yield	E+G+GE	0.0908(0.001)	0.9245(0.0023)
GBLUP	Maturity	E+G	0.0259(0.0002)	0.9181(0.001)
GBLUP	Maturity	E+G+GE	0.0256(0.0002)	0.9199(0.0008)
GBLUP	Height	E+G	0.0536(0.0006)	0.7674(0.0091)
GBLUP	Height	E+G+GE	0.0532(0.0006)	0.7711(0.0093)
GBLUP	Heading	E+G	0.0405(0.0006)	0.8683(0.006)
GBLUP	Heading	E+G+GE	0.0399(0.0006)	0.8725(0.0058)
GBLUP	Germination	E+G	0.082(0.0024)	0.5721(0.0141)
GBLUP	Germination	E+G+GE	0.082(0.0025)	0.5727(0.0142)
DL	Yield	E+G	0.094(0.0011)	0.9189(0.0032)
DL	Yield	E+G+GE	0.0923(0.0014)	0.922(0.0029)
DL	Maturity	E+G	0.0249(0.0002)	0.9249(0.0011)
DL	Maturity	E+G+GE	0.0252(0.0003)	0.9229(0.0012)
DL	Height	E+G	0.0545(0.0013)	0.7588(0.0163)
DL	Height	E+G+GE	0.0541(0.0006)	0.7628(0.0088)
DL	Heading	E+G	0.0376(0.0011)	0.8885(0.008)
DL	Heading	E+G+GE	0.0389(0.0008)	0.8798(0.0062)
DL	Germination	E+G	0.0823(0.0025)	0.5689(0.0166)
DL	Germination	E+G+GE	0.0825(0.0024)	0.5685(0.016)

SD represents the standard deviation of the metric across the folds.

For Maturity and Heading, the DL models demonstrated better performance under the two evaluated metrics; the GBLUP model yielded an average NRMSE between 1.6% and 7.68% higher compared to the values obtained with the DL models, and in terms of the average Pearson’s correlation (Cor), the DL models provided between 0.33% and 2.33% higher values compared to those obtained with the GBLUP model. Furthermore, we can observe the GBLUP model exhibited a slightly better performance in all traits when using the predictor that involved environment, line, and environment-line interaction effects (E+G+GE) compared to the predictor with only the first two effects (E+G). However, with DL, this situation was observed only for the traits Yield, Height, and Germination with NRMSE, and only for the first two of these traits (Yield, Height) with the Cor metric. This indicates the importance of the environment-line interaction effect in the mentioned traits.

We observed an overlap of the intervals formed by subtracting and adding one standard deviation (SD) to the average metric values obtained in each model for each trait and predictor. From this, we can infer a very similar performance of both evaluated models in the 5FCV strategy. In fact, the average values across the five traits and all predictors (E+G, E+G+GE) for the average metrics presented in **Table 2** are very similar, approximately 0.0587 for NRMSE and 0.81 for Cor.

### Tested lines in untested environments LOEO strategy

**Figure 4:**
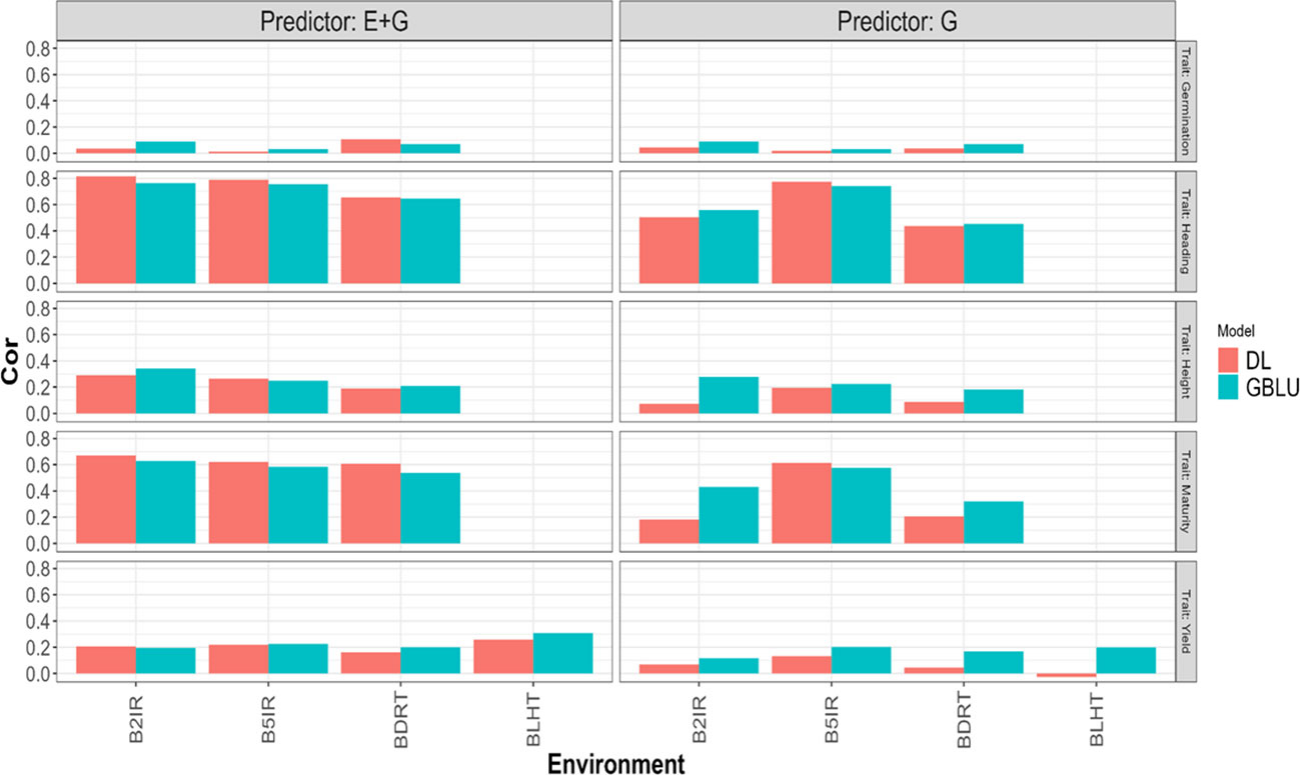
Pearson’s correlation obtained in each environment when applying LOEO strategy for each of the five traits (Germination, Heading, Height, Maturity, and Yield) for GBLUP and multi-modal deep learning (DL) models using two predictors (G and E+G).

**Figure 5:**
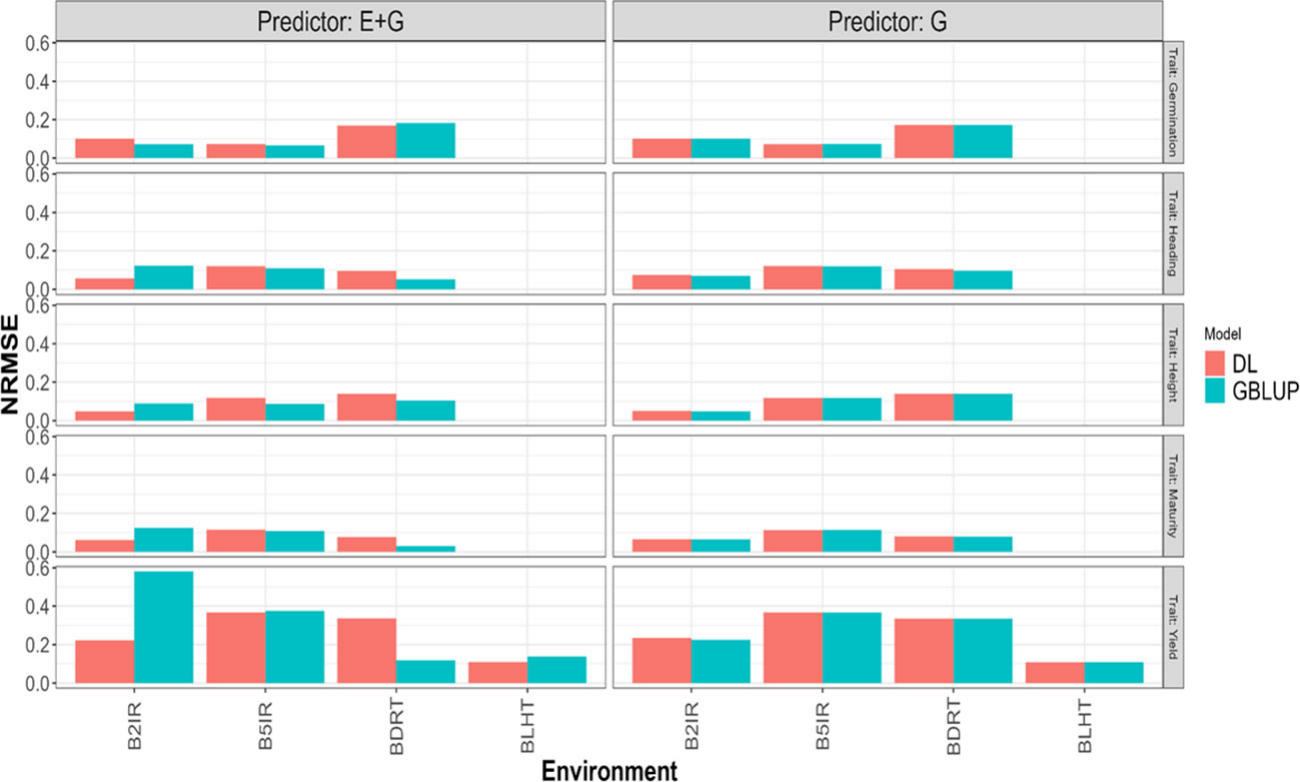
Normalized mean squared error (NRMSE) obtained in each environment when applying LOEO strategy for each of the five traits (Germination, Heading, Height, Maturity, and Yield) for GBLUP and multi-modal deep learning (DL) models using two predictors (G and E+G).

In the LOEO strategy, the information of a complete environment was predicted with the rest of the environments in each trait. This was done with the GBLUP Equation 1 and DL Equation 2 under two predictors, the first with only line effect (G) and the second with environment plus line effect (E+G). The results are presented in **Table 3** and **Figures 4**, **5**. The first column indicates the trait to be predicted, the second column represents the predictor used, the third column denotes the environment to predict, and the last two columns display the NRMSE and Cor values obtained with the GBLUP and DL models, respectively.

**Table 3:** Normalized root mean squared error of prediction (NRMSE) and average Pearson’s correlation (Cor) in LOEO evaluation strategy when predicting each one of the five traits (Yield, Maturity, Height, Heading and Germination) with GBLUP and DL models.

Trait	Model		GBLUP		DL	
	Predictor	Env	NRMSE	Cor	NRMSE	Cor
Yield	G	B2IR	**0.2244**	0.1151	0.2344	0.0688
Yield	E+G	B2IR	0.58	**0.196**	**0.2226**	**0.2072**
Yield	G	B5IR	**0.3678**	0.2025	**0.3679**	0.1323
Yield	E+G	B5IR	0.3757	**0.2242**	0.3679	**0.2183**
Yield	G	BDRT	0.3343	0.1682	**0.3345**	0.0444
Yield	E+G	BDRT	**0.1183**	**0.2004**	0.3366	**0.1612**
Yield	G	BLHT	**0.1087**	0.1979	**0.108**	-0.0262
Yield	E+G	BLHT	0.137	**0.3071**	0.1094	**0.259**
Maturity	G	B2IR	**0.065**	0.4312	0.066	0.1832
Maturity	E+G	B2IR	0.1242	**0.6294**	**0.0614**	**0.6697**
Maturity	G	B5IR	0.114	0.5775	**0.1133**	0.6127
Maturity	E+G	B5IR	**0.1092**	**0.5846**	0.1146	**0.6216**
Maturity	G	BDRT	0.0789	0.3197	0.0801	0.2058
Maturity	E+G	BDRT	**0.0307**	**0.5376**	**0.076**	**0.6061**
Height	G	B2IR	**0.0482**	0.2779	0.0502	0.0721
Height	E+G	B2IR	0.0888	**0.3433**	**0.0482**	**0.29**
Height	G	B5IR	0.1178	0.2243	**0.1171**	0.1943
Height	E+G	B5IR	**0.0873**	**0.2493**	0.1189	**0.2629**
Height	G	BDRT	0.1392	0.1805	**0.1392**	0.0875
Height	E+G	BDRT	**0.1047**	**0.2097**	0.1403	**0.1894**
Heading	G	B2IR	**0.0708**	0.5594	0.0754	0.5039
Heading	E+G	B2IR	0.1232	**0.7642**	**0.0568**	**0.8158**
Heading	G	B5IR	0.1197	0.7412	0.121	0.7732
Heading	E+G	B5IR	**0.1097**	**0.7558**	**0.1202**	**0.7894**
Heading	G	BDRT	0.097	0.4528	0.1051	0.4359
Heading	E+G	BDRT	**0.0512**	**0.6449**	**0.0953**	**0.6551**
Germination	G	B2IR	0.1004	**0.0907**	0.1012	**0.0447**
Germination	E+G	B2IR	**0.0723**	0.0895	**0.1002**	0.0352
Germination	G	B5IR	0.0735	0.0308	**0.0726**	**0.0184**
Germination	E+G	B5IR	**0.0651**	**0.0314**	0.073	0.0134
Germination	G	BDRT	**0.1727**	**0.0685**	0.1728	0.0357
Germination	E+G	BDRT	0.1823	0.068	**0.169**	**0.1063**

The best predictor (G or G+E) for each combination (model/trait) is indicated in bold.

Considering the 32 prediction scenarios, which correspond to all combinations of trait-predictor-environment (5 traits, 4 of these traits with three environments, and 1 trait with 4 environments, and 2 predictors (E and E+G)), we compared the performance of the models. In 11 out of 32 combinations, the DL model exhibited smaller NRMSE values, while in another 11 out of 32 combinations, the DL model achieved higher Pearson’s correlation values (Cor). Conversely, the GBLUP model outperformed the DL model in the remaining combinations.

### Yield

GBLUP and DL showed better Cor performance when using only the line effect (G) compared to the predictor E+G. However, the NRMSE results exhibited a different pattern. In the GBLUP model, the G predictor outperformed E+G in three out of the four environments (B2IR, B5IR, and BLTH), while for the DL model, the more complex predictor (E+G) was only better than G predictor in one environment (B2IR) out of four. For this trait, the DL model outperformed the GBLUP model in two out of the four predicted environments. Specifically, the DL model performed better than the GBLUP model in the BLTH environment when considering the NRMSE metric, and in the B2IR environment when considering the Cor metric.

### Maturity

GBLUP and DL showed better performance in terms of correlation (Cor) when using the E+G predictor compared to the G predictor. However, when considering the NRMSE metric, the results were opposite. The G predictor performed better in both models across all environments, exhibiting lower NRMSE values. Additionally, the DL model consistently showed higher correlation values than the GBLUP model in all environments. The DL model outperformed the GBLUP model in terms of NRMSE only in the B2IR environment.

### Height

The GBLUP model displayed better performance with the E+G predictor compared to the G predictor in two out of three environments for NRMSE and in all environments for Cor metric. However, the DL model exhibited a different pattern. For NRMSE, the G predictor outperformed E+G in two out of the three environments, while for Cor, the DL model achieved better performance with the E+G predictor in all environments. When comparing the models, the DL model showed better NRMSE performance in the B2IR environment, while the GBLUP model outperformed in the other environments. In terms of correlation (Cor), the DL model exhibited better performance in the B5IR environment, while in the rest of environments the GBLUP model was superior.

### Heading

The GBLUP model performed better with the E+G predictor compared to G in two out of the three environments for NRMSE and in all environments for Cor metrics. However, the DL model consistently showed better performance with the E+G predictor in terms of both NRMSE and Cor in all environments. In this case, the DL model outperformed the GBLUP model in all environments when considering the Cor metric, and for the NRMSE metric, the DL model was better in only one environment (B2IR).

### Germination

Both models showed better performance with the E+G predictor compared to the G predictor in two out of three environments in terms of NRMSE. However, the results were opposite in terms of Cor, where the G predictor exhibited better performance in the other two environments. In this case, the DL model outperformed the GBLUP model in the BDRT environment for both NRMSE and Cor metrics, and in the remaining two environments the GBLUP was better.

### Summary of the hyperparameter values used in the trained models

A summary of the optimal hyperparameter values used in the trained models for the 5FCV and LOEO evaluation strategies is provided in **Tables 4** and **5**. The descriptions of **Table 4** are:

- For the modality corresponding to environment effects (E), the optimal number of hidden layers more frequently found across the 5 partitions by the Bayesian optimization was 1 and 2 for models with predictor E+G and E+G+GE, respectively. This pattern was observed in the Germination and Height. In Heading and Maturity, the most frequently observed optimal number of hidden layers were 2 for the E+G predictor and 3 for the E+G+GE predictor. For Yield, the optimal number of hidden layers varied, with 1 being the most frequently observed for the E+G predictor, and 3 being the most frequently observed for the E+G+GE predictor. Regarding the optimal number of units, for Germination and Heigh, the most frequently observed values were 128 units for the E+G predictor and 89 units for the E+G+GE predictor. For Yield, Maturity, and Heading with predictor E+G+GE the units required were 60, and were 128, 114 and 114 for the same traits but under predictor E+G.
- For the modality corresponding to the Line effect (Z_L × L_G), the most frequently observed number of units was around 796 units for all traits in the model with the predictor E+G+GE. For the predictor E+G, the most frequently observed number of units varied across the traits, with 179, 183, 302, 302, and 472 units for the Yield, Height, Maturity, Heading, and Germination, respectively. Regarding the hidden layers in this modality, 3 and 1 were the most frequently observed values used in the models with both predictors (E+G and E+G+GE) for the Heading and Maturity traits. For the Height and Yield, regardless of the predictor (E+G and E+G+GE), the most frequently observed value was 1. Lastly, for Germination, the most frequently observed values for the number of hidden layers found by Bayesian optimization across the 5 partitions (5FCV) were 6 for the E+G predictor and 1 for the E+G+GE predictor.
- For the line-environment interaction modality effect, in all traits the most frequently optimal number of hidden layers observed was 1, and the corresponding optimal number of units varied depending on the trait. For Yield, Maturity, and Heading, the most frequently observed optimal number of units was 285, and for Germination and Height, the most frequently observed optimal number of units was 869.
- For 3 out of the 5 traits (Yield, Germination, and Height), in many of the folds, the DL model did not require hidden layers after the concatenation of the individual neural networks ( $n_{HLB_{2}}$ =0) when using the predictor E+G. In cases where more than one hidden layer was required, the most frequently observed optimal number of units ( $N_{2}$ ) was 200 and 100 for Yield, and approximately 100 for Height and Germination. For the other two traits, the required number of hidden layers was 3. For the model using the predictor E+G+GE, the most frequently observed number of hidden layers was 2 for three traits (Yield, Maturity, and Heading), and 1 for Germination and Height. For model with predictor E+G+GE, the more often hidden layers observed were 2 for traits Yield, Maturity and Heading, and for these three traits the most frequently optimal number of units was 32. For Germination and Height, the most frequently number of hidden layers used was 1 and the most frequently optimal number of units was 797.
- The most frequently optimal values for the patience hyperparameter (Pat) ranged between 1 and 128 across the 5 traits and the two evaluated predictors. The most observed value was 120. Regarding the rest of the hyperparameters, the regularization parameter (l) ranged between 0.0003 and 0.0088 across all traits and predictors, with an average optimal value of 0.004. The logarithm of the learning rate (llr), logarithm of the weight decay (lwd), and dropout regularization (DO) values ranged between (-7.4161, -4.6052), (-5.6717, -0.9163), and (0.0001, 0.3997) respectively. The average values of the most frequently observed values were -5.3167 for llr, -2.3874 for lwd, and 0.2117 for DO.

**Table 4:** Summary of the hyperparameter values used in the DL models for the 5-fold cross-validation (5FCV) performance evaluation strategy.

Trait	Predictor	l	llr	lwd	DO	$$N_{1}^{\left(1\right)}$$	$$N_{2}^{\left(1\right)}$$	$$N_{3}^{\left(1\right)}$$	$$N^{\left(1\right)}$$	$L_{1}$	$L_{2}$	$L_{3}$	L	Pat
Yield	E+G	0.0044	-4.8609	-0.9982	0.0046	1	1		0	128	179		200,100	1
Yield	E+G+GE	0.0003	-5.0740	-1.1720	0.1943	3	1	1	2	60	796	285	32	120
Maturity	E+G	0.0046	-7.4161	-5.6717	0.3997	2	3		3	114	302		76	42
Maturity	E+G+GE	0.0019	-5.5868	-1.5065	0.2521	3	1	1	2	60	796	285	32	120
Height	E+G	0.0078	-4.6052	-0.9163	0.0001	1	1		0	128	183		1	1
Height	E+G+GE	0.0045	-4.7462	-3.1049	0.3120	2	1	1	1	89	797	869	108	35
Heading	E+G	0.0023	-6.2648	-4.1796	0.1873	2	3		3	114	302		76	42
Heading	E+G+GE	0.0002	-5.1912	-1.2001	0.2250	3	1	1	2	60	796	285	32	120
Germination	E+G	0.0088	-4.6052	-0.9163	0.2001	1	6		0	128	472		200	128
Germination	E+G+GE	0.0075	-4.8166	-4.2082	0.3415	2	1	1	1	89	797	869	108	35

The first two columns indicate the trait and the predictor used in the evaluation. Columns 3 to 6 represent the average values of the regularization (l), the logarithm of the learning rate (llr), the logarithm of the weight decay (lwd) and the dropout rate (DO), respectively. In the columns 7 to 14 the most frequently observed optimal values (mode) across the 5 partitions in the 5-fold cross-validation (5FCV) for the hidden layers ( $L_{1},L_{2},L_{3}$ ) and the number of units ( $N_{1}^{\left(1\right)}$ , $N_{2}^{\left(1\right)},N_{3}^{\left(1\right)}$ ) in the respective networks for each modality in the model. These columns also include the information of the number of hidden layers ( L ) and number of units ( $N^{\left(1\right)}$ ) for the network created by concatenating the outputs of the individual networks before the output layer. The final column indicates the most frequently observed optimal value for the patience (Pat) hyperparameter registered in the early stopping criteria across the partitions.

**Table 5:** Summary of the hyperparameter values used in the DL models for the LOEO performance evaluation strategy.

Trait	Predictor	l	llr	lwd	DO	$L_{1}$	$L_{2}$	L	$$N_{1}^{\left(1\right)}$$	$$N_{1}^{\left(2\right)}$$	$$N^{\left(1\right)}$$	Pat
Yield	GID	0.0046	-5.6924	-6.8825	0.3364		5			552		51
Yield	GID+Env	0.0099	-4.6052	-0.9163	0.0001	1	1	0	128	183	107	1
Maturity	GID	0.0039	-7.0800	-6.6678	0.4942		1			709		30
Maturity	GID+Env	0.0030	-6.4791	-4.0866	0.2665	2	3	3	114	302	76	42
Height	GID	0.0094	-4.7878	-6.3700	0.2604		1			101		1
Height	GID+Env	0.0100	-4.6052	-0.9163	0.0001	4	1	4	128	905	140	128
Heading	GID	0.0085	-6.6059	-8.1575	0.2615		2			101		110
Heading	GID+Env	0.0015	-5.5421	-5.5715	0.1333	4	1	0, 3, 4	128	302	NA, 76, 119	37
Germination	GID	0.0028	-5.0766	-3.2896	0.2616		6			127		1
Germination	GID+Env	0.0064	-6.4791	-4.0866	0.2665	2	3	3	114	302	76	42

The first two columns indicate the trait and the predictor used in the evaluation. Columns 3 to 6 represent the average values of the regularization (l), the logarithm of the learning rate (llr), the logarithm of the weight decay (lwd) and the dropout rate (DO), respectively. In the first 6 columns of the last 7 columns correspond to the most frequently observed optimal values (mode) across the predicted environments for the hidden layers ( ${\text{L}}_{1},{\text{L}}_{2}$ ) and the number of units ( ${\text{N}}_{1}^{\left(1\right)}$ , ${\text{N}}_{2}^{\left(1\right)}$ ) in the respective networks for each modality in the model. In these columns also is include the information of the number of hidden layers ( L ) and number of units ( ${\text{N}}^{\left(1\right)}$ ) for the network created by concatenating the outputs of the individual networks before the output layer. The final column indicates the most frequently observed optimal value for the patience (Pat) hyperparameter registered in the early stopping criteria across the partitions.

When predicting a complete environment using the rest (LOEO), the most frequently optimal values of the integer hyperparameters (hidden layers, units, and patience) for the trained DL models are presented in **Table 5**. Additionally, the table includes the average values of the optimal real-valued hyperparameters (across environments) for the described Equation 2. While there are variations in the configurations of the NN models across traits and predictors, certain patterns can be observed. Across all traits and predictors, the average optimal values (across predicted environments) for the regularization parameter (l), the logarithm of the learning rate (llr), the logarithm of the weight decay (lwd), and dropout regularization (DO) fall within the intervals (0.0015, 0.01), (-7.08, -4.6051), (-8.1574, -0.9162), and (0.0001, 0.4942), respectively. The average values of these average optimal values are approximately in the middle of these intervals.

We observed the following patterns for the models with different predictors.

- For models with the predictor G, the most frequently optimal number of hidden layers (column $L_{2}$ ) for the corresponding neuronal networks were 5, 1, 1, 2, and 6 for traits Yield, Maturity, Height, Heading, and Germination, respectively. The corresponding number of units ( $N_{2}^{\left(1\right)})$ were 552, 709, 101, 101, and 127, with none reaching the upper bound of 1024 set in the search bounds (**Table 1**).
- For models with predictor E+G, in the individual NN of the modality of GID effect, the most frequently optimal number of hidden layers was 1 for Yield, Height, Heading, and 3 for traits Germination and Maturity. The corresponding most frequently optimal number of units used across the predicted environments were 183, 905, 302, 302, and 302 for Yield, Height, Germination, Maturity, and Heading, respectively.
- For models with the predictor E+G, in DL model with the modality corresponding to the Env effect (E), the most frequently optimal number of hidden layers were 4 in two traits (Heading and Height), 2 in two traits (Germination and Maturity), and 1 in the remaining trait (Yield). The corresponding most frequently optimal values of units were 128, 128, 114, 114, and 128 for traits Heading, Height, Germination, Maturity, and Yield, respectively. In Yield, no hidden layers were used in most of the fitted models after concatenating the outputs of the NNs of the involved inputs (Env and G), and for Heading were required 0, 3 and 4 hidden layers for the three predicted traits with none (not apply), 76 and 119 units, respectively. However, for Maturity, Height, and Germination, the most frequently optimal values for the number of hidden layers were 3, 4, and 3, respectively, as determined by the Bayesian optimization algorithm. When required at least one hidden layer ( $n_{HLB}>1$ ) in the trained model for predicting an environment, the most frequently optimal number of units for the first layer after concatenating the outputs of the individual NNs for Env and GID, were 107, 76, 140, 69, and 76 for Yield, Maturity, Height, Heading, and Germination, respectively.

### An impact evaluation of the data size on accuracy

An evaluation of the impact of the dataset size in the accuracy prediction but with less computational time was done using a reduced search space bounds as the specified in the shared code example. The search space includes the interval [1,2] for all hidden layers, [4,8] for the units of the environment effect, [32, 128] for the units in the line and effect, and the same interval for the units in the hidden layer for the MLP after concatenating the outputs of the neural networks of the two modalities (Environments and Lines effects). Additionally, we utilized the same search space for the rest of the hyperparameters, as described in **Table 1**.

This evaluation for both models (DL and GBLUP both with predictor E+G) was conducted by retaining 5%, 10%, 50%, 66.6%, and 80% (Percentage_tr) of the dataset for the training set, with the remainder used for the testing set. In all cases, we adhered to the spirit of the K-fold cross-validation strategy. For the first two cases (20-Fold and 10-Fold), the training and testing roles were inverted (1 fold for training and the rest of the folds for testing). For the last three cases, the traditional K-fold cross-validation strategy (2-Fold, 3-Fold, and 5-Fold) was implemented, where K-1 subsets were used for training, and the remaining subset was used for testing. Furthermore, the K-Folds in the third and fourth cases were repeated two times to obtain more representative results.

**Figure 6:**
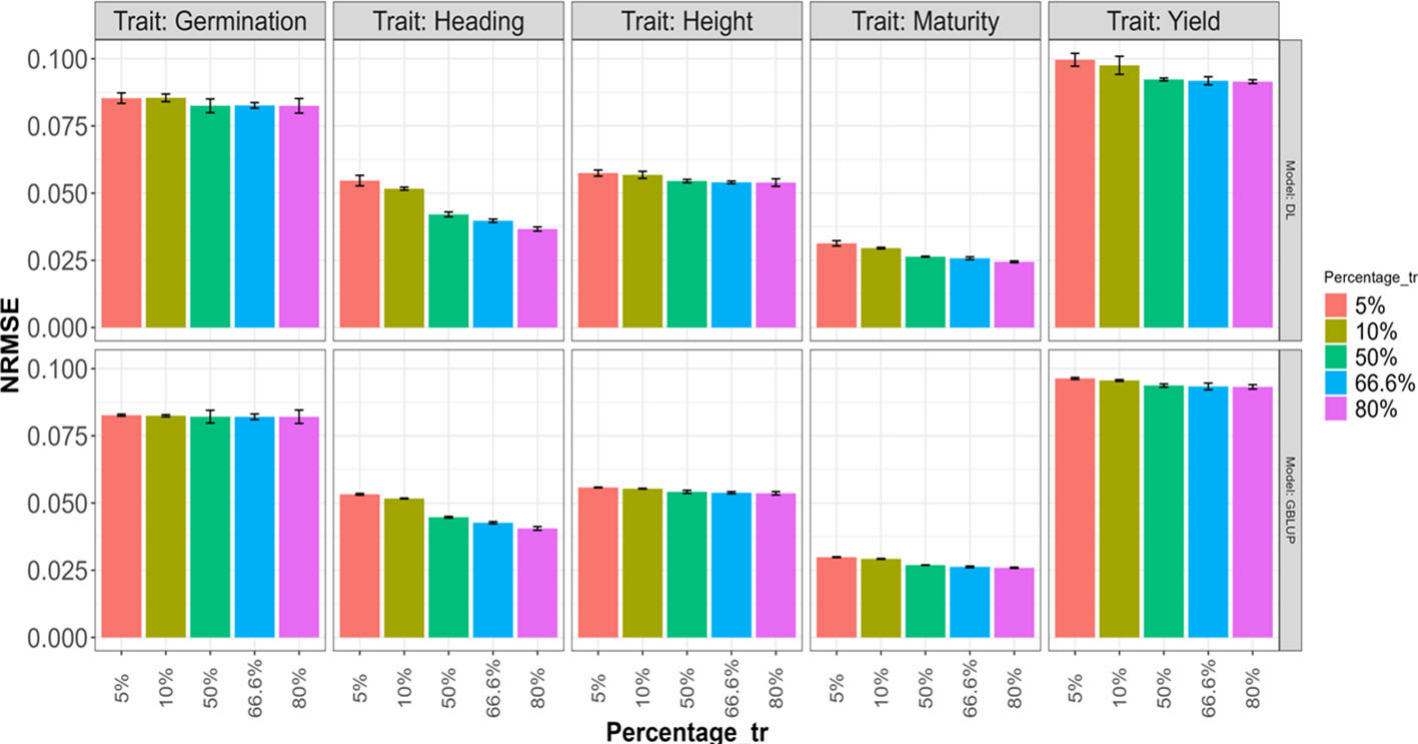
Average normalized mean squared error (NRMSE) across folds for each of the five traits (Germination, Heading, Height, Maturity, and Yield) for GBLUP and deep learning (DL) models using the predictor E+G. Percentages represent the portion of the dataset used for training. The bars for the first two values (5% and 10%) correspond to results in a 20-Fold and 10-Fold cross-validation strategy, with one-fold for training and the rest for testing. The remaining bars for the last three Percentage values correspond, respectively, to the traditional 2-Fold, 3-Fold, and 5-Fold cross-validation strategies, with the first two being repeated two times.

**Figure 7:**
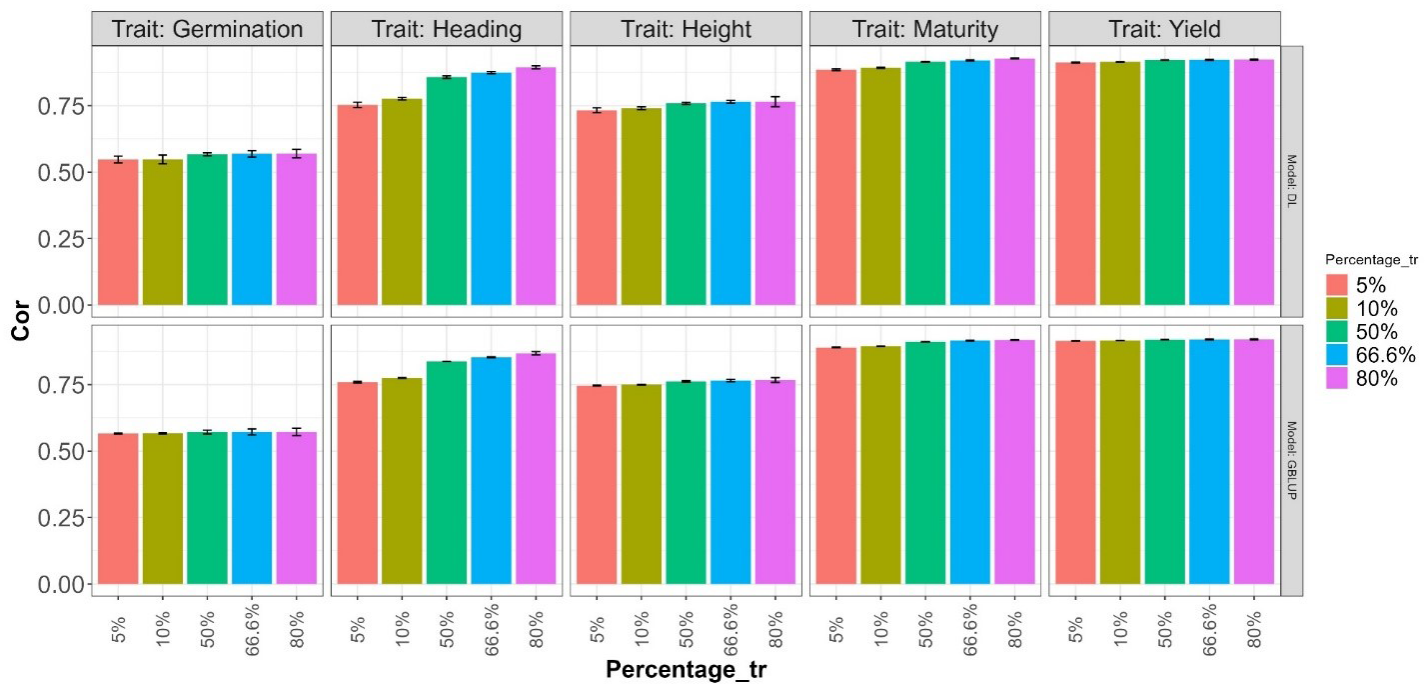
Average Pearson’s correlation (Cor) across folds for each of the five traits (Germination, Heading, Height, Maturity, and Yield) for GBLUP and deep learning (DL) models using the predictor E+G. Percentages represent the portion of the dataset used for training. The bars for the first two values (5% and 10%) correspond to results in a 20-Fold and 10-Fold cross-validation strategy, with one fold for training and the rest for testing. The remaining bars for the last three Percentage values correspond, respectively, to the traditional 2-Fold, 3-Fold, and 5-Fold cross-validation strategies, with the first two being repeated two times.

The obtained results are summarized in **Figures 6** and **7**, where the height of the bars represents the average metric values across folds. The vertical lines within each bar indicate the average minus and plus one standard deviation (SD) values of Cor obtained across folds. In the first of these figures (**Figure 6**), a deterioration in the normalized root mean squared error is observed as the training size decreases (moving right to left on the Percentage_tr axis) in both explored models. This deterioration is more pronounced in the Heading and Maturity traits. However, in all traits, this effect tends to be slightly smaller in the GBLUP model. A similar behavior is observed in **Figure 7** concerning the average Pearson’s correlation. These results are also very similar to those reported in the 5FCV strategy with the larger explored search space.

## Discussions

In this study, we utilized and expanded upon a recently proposed multi-modal DL model (Montesinos-López et al., 2023) for genomic prediction. Our extended model incorporated a neural network that takes as input the concatenated outputs of the individual NNs for each modality (E, G, and GE, for example). The improved performance of the DL models can be attributed, in part, to the novel architecture employed and to the availability of a moderately larger dataset.

Within the application of multi-modal deep learning in the context of genomic selection, it is important to take advantages of the virtues of multi-modal deep learning:

(1) Enhanced representation learning, by integrating different modalities, since multi-modal deep learning can learn richer and more comprehensive representations of data. This allows for a more holistic understanding of the input, capturing both complementary and redundant information across modalities.
(2) Improved performance because multi-modal deep learning outperforms single-modal approaches in various tasks, including image captioning, video understanding, speech recognition, and more. By leveraging multiple modalities, the model can exploit the strengths of each modality to improve overall performance.
(3) Robustness to data limitations because multi-modal learning can mitigate the limitations of individual modalities by leveraging complementary information. If one modality lacks sufficient data or exhibits noise or ambiguity, the model can rely on other modalities to compensate for these shortcomings, resulting in improved robustness and generalization.
(4) Richer context understanding, since combining different modalities allows for a more comprehensive understanding of context. For example, in natural language processing tasks, incorporating visual information alongside text can provide valuable visual context that enhances language understanding and generates more accurate responses.
(5) Cross-modal transfer learning since multi-modal deep learning models can transfer knowledge between different modalities. Pretraining on one modality and fine-tuning on another can accelerate the learning process and improve performance, even with limited labeled data in the target modality.
(6) Better human-like perception, since humans naturally integrate information from multiple senses to perceive and interpret the world. Multi-modal deep learning aims to mimic this human-like perception by fusing information from diverse modalities, enabling machines to understand and interact with the environment in a more human-centric way.
(7) Discovering hidden relationships because multi-modal learning can uncover hidden relationships and correlations between different modalities that may not be apparent in isolation. This can lead to new insights and discoveries, especially in domains where the data is inherently multi-modal, such as in healthcare, autonomous driving, and social media analysis.

These virtues make multi-modal deep learning a promising approach for a wide range of tasks and domains, allowing for richer and more nuanced data analysis, understanding, and decision-making and our findings provide further evidence of the competitiveness of multi-model deep learning models, particularly when leveraging more sophisticated architectures that incorporate late fusion strategies (Ramachandram and Taylor, 2017; Baltrušaitis et al., 2018), as seen in the extension of the model used by Montesinos-López et al. (2023). Additionally, our study benefits from the utilization of larger datasets.

The results of our study demonstrate the multi-modal DL models proposed outperform GBLUP models in certain traits and exhibit similar performance in others. However, when predicting for an entire year, the performance, while still comparable, is slightly reduced compared to the GBLUP model. This could be attributed to the relatively smaller training size available for the models in these scenarios, in which more exploration can be done where other strategy tuning parameters and loss function could be evaluated.

Our results agree with the growing evidence that multi-modal deep learning models are a powerful tool for predicting more efficiently in the context where multiple-inputs capture different portions of the signal of the response variable. Because the modelling process trains a particular deep neural network for each input (modality), at the end, all the outputs of these deep neural networks are concatenated in a final deep neural network that produces the final predictions. The multi-modal deep learning for its architecture (**Figure 1**) facilitates the training process to efficiently capture the signal of the response and control of the overfitting. For these reasons, application of multi-modal deep learning models continues growing in many fields like health care, bioinformatics, computer vision, etc.

Finally, it is important to note that by leveraging the power of multi-modal deep learning, genomic prediction can benefit from the integration of diverse data sources, improved prediction accuracy, robustness to missing data, and enhanced interpretability, ultimately advancing our understanding of genetic traits and their implications in various applications, including precision medicine and agricultural breeding programs.

## Conclusions

Using a moderately large dataset comprising 4464 lines evaluated for 5 agronomic traits under 3 or 4 different environments, we conducted a comparative analysis between GBLUP models implemented in the BGLR R package and a novel multi-modal deep learning (DL) model developed in this study. The results demonstrate the extended DL model presented achieved higher accuracy in predicting certain traits, specifically Maturity and Heading, when evaluated using the 5FCV. The DL model exhibited comparable accuracy to the GBLUP models for the remaining traits: Yield, Height, and Germination.

The DL approach utilized in this study extends and complements the previously proposed model, resulting in significant improvements in prediction accuracy for new environments. This finding further supports the notion that constructing individual networks for each modality and subsequently combining their outputs to feed into another network can yield more flexible and accurate models.

## Data availability statement

Publicly available datasets were analyzed in this study. The phenotypic and genomic wheat data employed in this study can be downloaded from the following link https://hdl.handle.net/11529/10548813 (Montesinos-López et al., 2023).

## Author contributions

JC: Conceptualization, Investigation, Writing – review & editing. AM-L: Conceptualization, Investigation, Methodology, Software, Writing – original draft, Writing – review & editing. LC-H: Data curation, Project administration, Writing – review & editing. SD: Investigation, Methodology, Writing – review & editing. GG: Conceptualization, Validation, Writing – review & editing. PV: Data curation, Visualization, Writing – review and editing. CS: Data curation, Funding acquisition, Supervision, Writing – review & editing. VG: Data curation, Validation, Writing – review & editing. ZT: Data curation, Investigation, Visualization, Writing – review & editing. MF: Software, Writing – review & editing. PP-R: Validation, Writing – review & editing. SR-P: Investigation, Software, Writing – review & editing. ML: Conceptualization, Methodology, Writing – review & editing. HL: Conceptualization, Methodology, Writing – review & editing. OM-L: Conceptualization, Formal Analysis, Investigation, Methodology, Software, Writing – review & editing.
